# Electromagnetic Field Effect or Simply Stress? Effects of UMTS Exposure on Hippocampal Longterm Plasticity in the Context of Procedure Related Hormone Release

**DOI:** 10.1371/journal.pone.0019437

**Published:** 2011-05-05

**Authors:** Nora Prochnow, Tina Gebing, Kerstin Ladage, Dorothee Krause-Finkeldey, Abdessamad El Ouardi, Andreas Bitz, Joachim Streckert, Volkert Hansen, Rolf Dermietzel

**Affiliations:** 1 Department of Neuroanatomy and Molecular Brain Research, Ruhr-University Bochum, Bochum, Germany; 2 Chair of Electromagnetic Theory, University of Wuppertal, Wuppertal, Germany; University of Muenster, Germany

## Abstract

Harmful effects of electromagnetic fields (EMF) on cognitive and behavioural features of humans and rodents have been controversially discussed and raised persistent concern about adverse effects of EMF on general brain functions. In the present study we applied radio-frequency (RF) signals of the Universal Mobile Telecommunications System (UMTS) to full brain exposed male Wistar rats in order to elaborate putative influences on stress hormone release (corticosteron; CORT and adrenocorticotropic hormone; ACTH) and on hippocampal derived synaptic long-term plasticity (LTP) and depression (LTD) as electrophysiological hallmarks for memory storage and memory consolidation. Exposure was computer controlled providing blind conditions. Nominal brain-averaged specific absorption rates (SAR) as a measure of applied mass-related dissipated RF power were 0, 2, and 10 W/kg over a period of 120 min. Comparison of cage exposed animals revealed, regardless of EMF exposure, significantly increased CORT and ACTH levels which corresponded with generally decreased field potential slopes and amplitudes in hippocampal LTP and LTD. Animals following SAR exposure of 2 W/kg (averaged over the whole brain of 2.3 g tissue mass) did not differ from the sham-exposed group in LTP and LTD experiments. In contrast, a significant reduction in LTP and LTD was observed at the high power rate of SAR (10 W/kg). The results demonstrate that a rate of 2 W/kg displays no adverse impact on LTP and LTD, while 10 W/kg leads to significant effects on the electrophysiological parameters, which can be clearly distinguished from the stress derived background. Our findings suggest that UMTS exposure with SAR in the range of 2 W/kg is not harmful to critical markers for memory storage and memory consolidation, however, an influence of UMTS at high energy absorption rates (10 W/kg) cannot be excluded.

## Introduction

The current increase in daily mobile phone use has caused consistent concern regarding putative adverse influences of radio-frequency (RF) electromagnetic fields (EMF) emitted from present telecommunication systems on human health. Until this time most studies focussed on the 2nd generation Global System for Mobile Communications with operation frequencies (in Europe) between 880 and 960 MHz or between 1710 and 1880 MHz, respectively, but only a few concentrated on the investigation of possible effects of the more recently introduced Universal Mobile Telecommunications System UMTS (1.92 to 2.17 GHz), which belongs to the third generation of mobile communication standards.

With the help of specifically designed exposure systems it could be repeatedly shown that UMTS exposure did not reveal significant changes between sham and field exposed animals, but in turn exposure-conditioned groups most often differed significantly from the related cage controls. This is particularly evident when studies related to effects of EMF on brain functions are considered.

Zwamborn et al. [1] described a reduction of well-being in humans after base station-like exposure in subjects with and without self-reported sensitivity to EMF. In contrast to these initial data on humans Regel et al. [2] focused on event-related potentials, cognitive functions, well-being and vigilance-controlled electroencephalograms and were unable to show significant changes compared to the sham group, findings which were corroborated in follow-up studies by Kleinlogel et al. [3] and Unterlechner et al. [4]. Likewise, in individuals reporting sensitivity to EMF no changes in cognitive functioning or physiological measures compared to controls were reported after short-term exposure to UMTS base station signals [5]. Although there was an increase in ‘headache rating’, comparisons between adult and adolescent subjects who had previously been exposed to UMTS sources for 45 minutes revealed no changes for the outcome in cognitive tasks [6]. Regarding the stronger but much more localized exposure by mobile phone headsets there is still a controversial discussion concerning non-thermal effects on humans (for review see [7]).

The role of EMF effects on neuronal mechanisms like synaptic efficacy in the central nervous system, which can be regarded as a neurophysiological correlate of higher brain functions has also been discussed. Up to now there exists only a single study related to effects of field exposure on synaptic plasticity in the rat hippocampus, a brain region found to be essential for memory storage and consolidation. Hippocampal slices revealed a decrease in basic synaptic activity when treated directly with an *ex vivo*-EMF exposure to 50 Hz magnetic fields [8].

A major handicap in terms of comparability of available animal studies is their variance in applied protocols, i.e. whole body exposure versus local head exposure, lack of assessment of thermal impact on exposed brain structures, etc. Also, exposure system derived stress, as applied due to the animal's body restraint during full brain exposure, has consistently been neglected.

In this study we describe a new application device that allows RF exposure in an almost stereotactical manner in rat, meaning in a local restricted and controllable fashion. Furthermore, we took care of monitoring stress-induced hormone release by assessing adrenocorticotropic hormone (ACTH) and corticosteron (CORT) levels as major hallmarks of stress-activated hypothalamic-pituitary-adrenal axis (HPA).

The essence of the study indicates that besides considerable activation of the HPA through the restraint conditions EMF gained significant effects on LTD and LTP, and on basic synaptic transmission only for exposures of excessive specific absorption rates (10 W/kg).

## Materials and Methods

### Ethical statement

All animal experimentation described here, were carried out in accordance with the regulations set down by the international guidelines for proper handling of experimental animals and by the Federal Republic of Germany, and were specifically approved by the local authorities (Landesamt für Natur, Umwelt und Verbraucherschutz Nordrhein-Westfalen, Germany; experimental contract approval No. 8.87-50.10.32.09.098).

### Animals

Male Wistar albino rats (12–15 weeks old and approximately 200 g) were used in all experiments. Male animals were chosen to avoid oestrus dependent effects on stress responses or hippocampal plasticity. Prior to all experimental settings, animals were subjected to the same conditions: Rats were housed in groups of 4 animals per cage in a temperature controlled room (22°C) under artificial illumination (12∶12 light/dark cycle; lit on 07:00 a.m.). Water and food was given *ad libitum*. For familiarization and to reduce restraint and contact stress in advance to the EMF/sham exposure, an open restraint tube was added to each cage during the full housing period of the animals. All subjects were handled daily by the same researcher from weaning until execution or exposure to the EMF system, to minimize stress or adverse reactions to manipulation. Handling consisted of picking up the animal from its home cage by placing the hand over the animal's back, with thumb and forefinger pressing its forelegs towards its head. Then the animal was placed into the empty gassing chamber (8 min duration; start 11 a.m.) and finally replacing it into its home cage. At the day of experiment or sacrifice the animals were handled in the same manner with the exception that the gassing chamber contained isoflourane. Isoflourane treatment was used to reduce handling stress due to the insertion into the restraint tubes or into the guillotine. Insertion into the tubes as well as into the EMF system were performed in a fast and noise free routine to avoid awakening.

Asides the described handling procedure three rats were trained to enter the guillotine on their own. These animals were sacrificed without presentiment and served as “zero-stress” controls. The final groups of experimental animals were grouped according to **Table 1**.

**Table 1:** Investigated groups and terminology.

Terminus	Explanation/Condition	Restraint
ED-only	Exposure device only, Sham treated animals	**+**
ED-EMF	Exposure device, SAR 2 and 10 W/kg exposure	**+**
Cage controls	Unexposed free running animals without ED visit	**-**
Handled rats	“zero-stress” controls for plasma hormone measurements	**-**

All EMF related experimental procedures were performed between 9:00 and 12:00 a.m. in order to avoid unwanted fluctuations in blood concentrations of the hormone levels linked to diurnal rhythm.

### Electromagnetic field exposure

An approved concept for the RF head exposure of a number of animals is the arrangement of the restrained subjects on a circle around a common source, e.g. using a carousel [9] or a radial waveguide [10] set-up, and to align the snouts towards the feed. In case of brain exposure, such a concept has been criticized by experts [11], arguing that the sensitive sensors around the rodents' snouts could be overexposed, thus masking the expected small effects on the brain. Another concept, which avoids the latter restraints is to place an antenna directly onto the head of the rat [12]. Nevertheless, the use of such an open structure is disadvantageous, if a number of animals are to be exposed, because of electromagnetic coupling between adjacent antennas.

**Figure 1:**
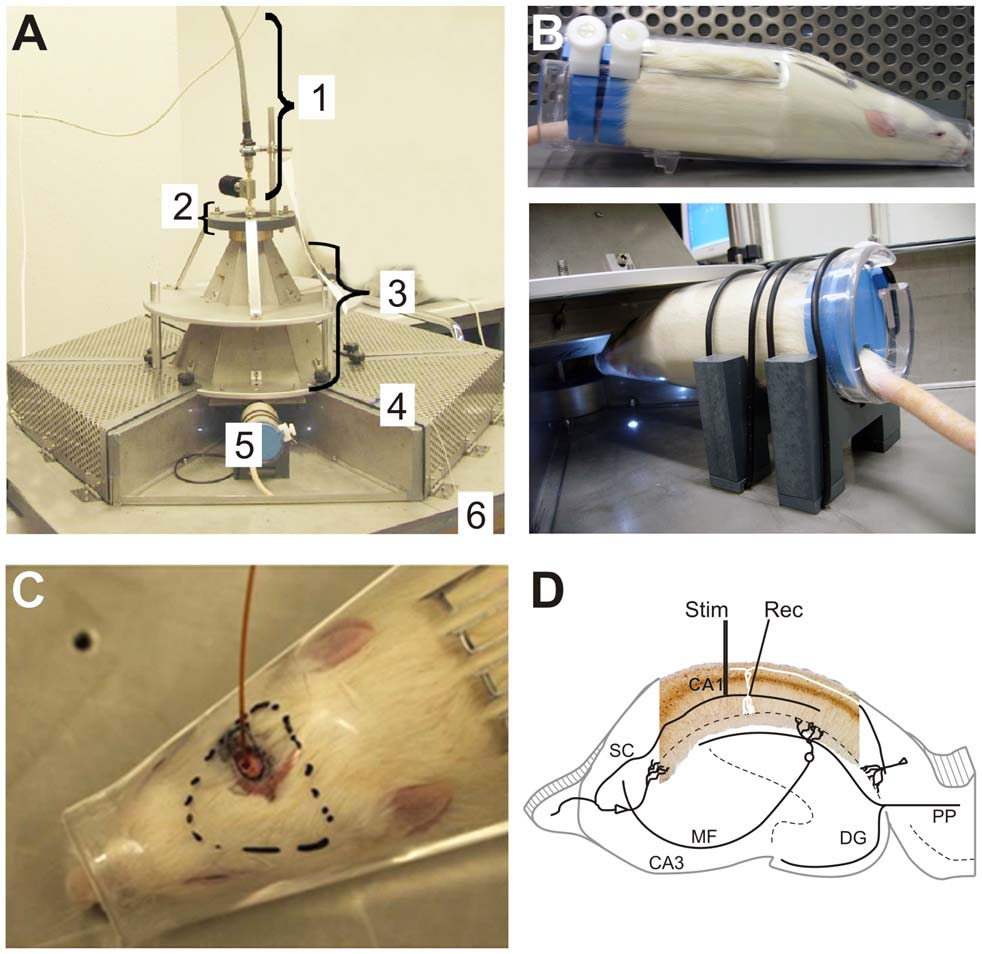
Technical implementations of full brain UMTS exposure. Figures **1A–C** represent the technical implementation of the full-brain UMTS exposure device for six 12–15 weeks aged male Wistar rats. **1A** depicts the complete exposure device with 1) the feed, 2) a 1:6 power splitter, 3) the hexagonal double-cone waveguide, 4) 6 shielded exposure compartments, 5) 6 restrainers with holders, 6) the ground plate. Prior to electromagnetic field exposure, animals were inserted into cone-shaped body restrainers to avoid unwanted head and/or body movements (**1B**, upper image) and subsequently placed and fixed under the EMF exposure cone (**1B**, lower image). Note that the brain of the animal within the restrainer is located below the lower end of the pair of metal ribs built into each compartment of the double-cone waveguide. **1C** is a photograph of the rat cadaver in the restrainer with the fibre-optic temperature probe contacted to the brain tissue through a bore in the cranial bone. Fig. **1D** represents the recording and stimulus electrode position for excitatory field potential (fEPSP) recordings of CA1 pyramidal cells: Electrical stimulation (Stim) was applied to the *Schaffer* collateral pathway to elicit fEPSPs on the dendritic site of CA1 neurons. LTP was evoked by using high frequency stimulation (HFS) with four trains of 10 shocks at 100 Hz every 1 sec. Low frequency stimulation (LFS) for LTD recording was set to one trail of 900 shocks at 1 Hz.

In order to overcome these problems, we modified the concept of the flat radial waveguide and transformed it into a spherical sector waveguide [13]. Since 6 restrained rats should be exposed simultaneously, the spherical waveguide was approached by a double-cone waveguide, consisting of an inner and an outer cone of hexagonal bases, with a coaxial feed at the tip and with the exposure field leaking from the open end at the bottom (**Fig. 1A**). In an integrated 1∶6 power splitter on top of the hexagonal structure, the input power is uniformly distributed over the six compartments of the waveguide. Since the exposure field should be concentrated to a rather small area at the lower aperture of the double-cone, metal ribs fixed to the opposite walls of the inner and outer cone, respectively, were built into each compartment with a gap of 6 mm in between. The 6 conic restrainers (**Fig. 1B** top) for the immobilisation of the rats are fixed in such a way that the rats' brains are always positioned directly below the lower end of the gap between the ribs (**Fig. 1B** bottom). In order to avoid electromagnetic coupling between the exposure fields of adjacent animals, the restrainers are placed into compartments which are shielded against each other and against the environment by using metal walls and hinged flaps covered with finely woven metal grids. The size of the ground plane is 1.1 m ×1.1 m, the height from the ground plane to the tip is 40 cm.

The 2-GHz signal source consists of a generator delivering the UMTS signal defined in Ndoumbè et al. [14] and of a booster amplifier, whereby a computerized experimental protocol provides a blinded scrambling of the sequentially applied specific absorption rates in the rat brain (0 W/kg, 2 W/kg and 10 W/kg). The actual input power level is monitored via a coupler/detector combination.

During the 2 hours of nonstop exposure animals were kept at daylight condition and laminar air flow to avoid distraction by spatial or olfactory cues.

The objective of the study design was a (blinded) multi-level exposure (including sham-exposure) with the highest SAR lying below but close to the thermal limit, i.e. the maximum applied SAR should not provoke a temperature elevation inside the rat's brain (ΔT<0.1°C). In contrast to the situation of human exposure neither recommendations of standardization bodies nor reliable literature data for the dosimetry in RF-exposed rat brains are available. Therefore, an experimental dosimetry using rat cadavers and narcotized rats (with Ketamin (100 mg/kg) Xylazine hydrochloride 2% (5 mg/kg and Chloralhydrate 200 mg/kg intraperitoneally) was performed in an own pre-study in order to find appropriate SAR values. To do so, a thin metal-free fibre-optic temperature probe (FISO FOT-M) was inserted on a length of 1.6 cm into the brain of the subjects through a tiny bore in the cranial bone (**Fig. 1C**) and temperature change was recorded during application of the RF field.

### Slice preparation and extracellular recording

For slice preparations animals were deeply anaesthetized with isoflurane and then killed by decapitation. Hippocampal horizontal slices (400 µm thick) were cut with a Leica VT1000 vibratome (Leica Microsystems, Wetzlar, Germany) in ice cold artificial cerebrospinal fluid (ACSF) containing a high concentration of sucrose (in mM): 85 NaCl, 25 NaHCO_3_, 2.5 KCl, 4 MgSO_4_, 0.5 CaCl_2_, 1.25 NaH_2_PO_4_, 25 Glucose, 7.5 Sucrose, 0.5 Ascorbic acid) and then incubated for at least 1 h prior to recording in room temperatured normal ACSF containing in mM: 119 NaCl, 26 NaHCO_3_, 2.5 KCl, 1.3 MgSO_4_, 2.5 CaCl_2_, 1 NaH_2_PO_4_, 10 Glucose. External solutions were continuously gassed with 95% O_2_/5% CO_2_. All slices were cut in high sucrose ACSF. After recovery in normal ACSF slices were transferred to a recording chamber mounted on a fixed stage ZEISS Axioscope (Carl Zeiss AG, Goettingen Germany) continuosly superfused (3–4 ml/min) with room temperatured and oxigenized normal ACSF. For intensified visualization of the tissue a *Dodt* Gradient Contrast Filter (Luigs & Neumann, Ratingen, Germany) was used.

Field excitatory postsynaptic potentials (fEPSPs) were recorded extracellularly by placing low resistance (2–5 MΩ) borosilicate patch pipettes (Glass Capillary Tubing, Corning 7058; Hugo Sachs Elektronik, Harvard Apparatus; March-Hugstetten, Germany) filled with 1 M NaCl into the stratum radiatum. A concentric SNEX1200 Wolfram electrode (Hugo Sachs Elektronik, Harvard Apparatus, March-Hugstetten, Germany) was placed into the *Schaffer* collateral fibers to stimulate inputs to hippocampal CA1 pyramidal neurons (**Fig. 1D**). fEPSP and population spike responses were driven by bipolar stimuli (*150–400 µA*) with a STG 1200 (Multi channel systems, Reutlingen, Germany). Before recording, input/output curves were calculated to set the optimal stimulation intensity which was adjusted to 50% of the evoked maximal response amplitude. To elicit fEPSP response 20 min of 5 Hz baseline stimulation was performed in advance to the applied LTP or LTD protocol. LTP was evoked by using high frequency stimulation (HFS) with four trains of 10 shocks at 100 Hz every 1 sec [15]. Low frequency stimulation (LFS) for LTD recording was set to 900 shocks at 1 Hz [16].

All responses were amplified and filtered by an Axopatch 200B amplifier (Axon instruments; Molecular Devices, Sunnyvale CA, USA), digitized at 20 kHz and displayed, stored and analyzed using WinWCP software (Strathclyde; Biologic, Knoxville TN, USA). A 16 bit analog to digital converter (BNC 2110 connected to Ni-PCi 6229; National Instruments; Munich, Germany) was used to digitize the signals.

### Data analysis

All fEPSP data were normalized as a percent of control, based on average amplitudes from the 20 min recording immediately before LTP or LTD protocol application. For each time point, 2 consecutive responses at 20 sec intervals were averaged and the results were expressed as the mean percentage ± standard error of the mean (SEM).

Sigma Plot 11.0 (Systat Software, Chicago, USA) was used to compare and visualize stress and electromagnetic field induced effects between groups of exposure matched experiments (oneway analysis of variance; ANOVA).

Holm-Sidak post-hoc tests [17] were utilized to examine group differences of data derived from LTP and LTD experiments following significant omnibus ANOVAs.

Stress related plasma hormone level statistics was performed via ANOVA and subsequent Newman-Keuls analysis of the obtained data according to Foy et al. [18]. Alpha was set at 0.05 and outlier data points greater than 3 standard deviations from exclusive group means were eliminated from analyses.

All values are expressed as mean ± standard error of the mean (SEM). The level of significance was set at p<0.05 [p<0.05 =  _*_; p<0.005 =  _**_; p<0.0005 =  _***_].

### Essays of stress related hormones

Full blood samples of each animal were taken for serum analysis at the time of sacrifice according to Klemm and Gupta [19]. Trunk blood was stored in heparinised tubes on ice until brain preparations were finished. Then, plasma and serum were attained after 20 min centrifugation at 4000 rpm at 4°C. Individual samples were frozen and stored at −80°C for subsequent determination of adrenocorticotropic hormone (ACTH) and corticosteron (CORT). Concentration measurements of both factors were performed by LABOKLIN (Bad Kissingen, Germany) using specific radioimmunoessays.

## Results

### Field and SAR distribution

**Figure 2:**
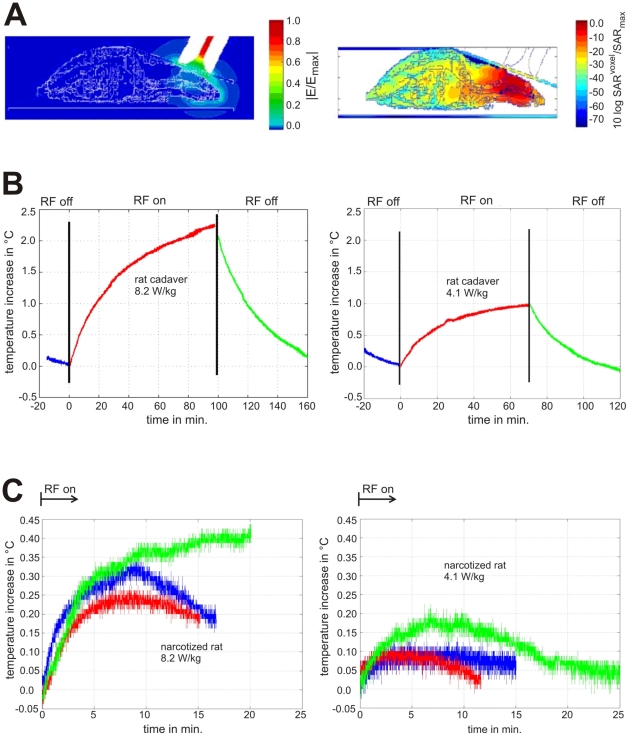
Results of the numerical and experimental dosimetry concerning the RF exposure. Figure **2A–C** shows results of the numerical and experimental dosimetry in order to characterize the RF exposure. Fig. **2A** gives computed results of the electrical field (**2A**, left image) and SAR distributions (**2A**, right image) indicating the local character of the rat's RF exposure. In Figs. **2B** and **2C** the temperature development measured during the pre-study with the fibre-optic temperature probe placed in the brain (Fig. 1C) of rat cadavers and of narcotized animals, respectively, is displayed for 2.3 g-brain-averaged SAR values of 8.2 (left images) and 4.1 W/kg (right images). Each of the curves in Fig. **2B** which were achieved with dead animals represents a typical temperature response of 1 individual animal. Long times of investigation with the possibility to observe the reaction of switching the RF field on and off were no problem here due to the use of cadavers. The temperature development shows the expected behavior of passive lossy dielectric bodies with heat convection through its surface as main contribution leading to a steady-state. On the other hand, each of the 6 curves in Figure **2C** represents the development of temperature in the brain of an anesthetized individual male, adult Wistar rat after onset of RF exposure. It is assumed that the differing dynamic of the temperature curves is due to different depths of the anesthetical state of the animals.

Due to the specific design of the exposure device a very localized exposure is achieved. The normalized electrical field and SAR distributions in the cuts shown in **fig. 2A** demonstrate that the exposure concentrates mainly on the rat's brain. Part of the electromagnetic field is reflected into the waveguide. The rest is absorbed in the rats' bodies, therefore the power scattered to the environment is negligibly small. The max/min-ratio of the SAR across the six exposure places was assessed from electrical field measurements as only 1.19.

### Thermal behavior

From the pre-study concerning thermal aspects of the RF exposure typical individual results are given for the temperature development in rat cadavers (**Fig. 2B**) and in narcotized animals (**Fig. 2C**), following the onset of the RF signal both for brain-averaged SAR values of 8.2 and 4.1 W/kg, respectively (relating to an input power of 1.4 and 0.7 W for the double-cone waveguide occupied with 1 instead of 6 animals). On the basis of computer models of adult Wistar rats an approach to the permitted non-thermal SAR limit was numerically found around 8 W/kg (2.3 g brain-averaged) and the SAR of 8.2 W/kg was therefore used here as the maximum starting value. The curves show a rather long-lasting temperature increase in dead rats and a substantially reduced temperature effect in narcotized rats due to the blood flow and due to the active though still damped thermal regulation [20], [21], [22], [23]. Since only two parameters are important (1. the starting slope of the temperature curve being proportional to SAR, and 2. the temperature maximum), the measurements with narcotized animals were terminated when a stabilization of the curves became obvious. While the unambiguous initial temperature slopes in **fig. 2B** allow a determination of the SAR by differentiation (SAR  =  c (ΔT/Δt)|_t = 0_, with c being the specific heat capacity in J/(kg K)), this is not trustworthy for the rather noisy curves of **fig. 2C**. It reveals that in either case both applied SAR values lead to an initial increase (<10 min.), followed by a stabilization or decay in brain tissue temperature. It turned out that the longer the exposure duration, the less is the effect per time, although the animals were deeply anesthetized. The finding of a saturation curve is indicating stabilization of the process after reaching a time- and SAR-dependent threshold.

Therefore, with regard to the main experiment we did not expect a 2 hours lasting exposure leading to effects, which could not be compensated by the nervous system's intrinsic thermo-autoregulatory mechanisms in the awake animal [24]. Furthermore, the obtained data for 8 W/kg during anaesthesia only revealed temperature shifts between 0.2 and 0.4°C which are still 2.5 to 5 times less compared to the basic core temperature fluctuation of a rat under resting conditions [25]. The applied Chloralhydrate is described to already elicit a body-core/brain temperature differential of 1.8°C [22] in addition to the expected effects by Ketanest/Xylazine (>2°C; [26], [27]). The uncompensated temperature shift in the anesthetized animals in our experiments evoked by 4.1 W/kg ranged between 0.05 and 0.2°C in maximum and evoked by 8.2 W/kg ranged between 0.2 and 0.4°C. The measured changes still represent a fractional amount of the temperature differential, elicited by narcotization. Thus, it was decided with regard to the expected more effective thermo-regulation in non-anesthetized animals to perform the main experiment with a 25%-increased maximum SAR of 10 W/kg (brain-avg.). To produce a brain-averaged SAR of 10 W/kg in any of the six rats placed under the double-cone waveguide at one time, a total input power of 1.8 W had to be applied.

**Table 2** summarizes the obvious findings. It reveals a reasonable good coincidence of experimentally and numerically derived SAR or temperature increase for the rat cadaver and for the narcotized rat, respectively. The exposure of cadavers must be classified as thermal and of narcotized animals as definitely non-thermal (at 4.1 W/kg) or at the most slightly thermal (at 8.2 W/kg). For the main experiments one has to consider that in vital non-narcotized rats the thermal regulatory system is fully activated. Thus, on the basis of the measurements and calculations performed for cadavers and narcotized animals a power adjustment of the exposure device giving rise to SARs of 2W/kg excludes thermal overload of the animals and to 10 W/kg is certainly suitable.

**Table 2:** Results of the pre-study for brain exposure investigation.

	Pre-study				Main experiment		
	rat cadavers		narcotized rats		vital rats		
**Input power**	1.4 W*)	0.7 W*)	1.4 W*)	0.7 W*)	1.8 W	0.36 W	0 W
**No. of animals**	n = 6	n = 3	n = 3		n = 6		
**ΔT_brain_ (meas.)**	2.16°C±10%	0.85°C±21%	0.2–0.4°C** ^)^	0.05–0.2°C** ^)^	-	-	-
**ΔT_brain_ (calc.)**	-	-	0.3°C		-	-	-
**SAR_brain_**	7.75 W/kg±18%	3.9 W/kg±23%	-	-	-	-	-
**(meas.)** ^***)^							
**SAR_brain_ (cal.)**	8.2 W/kg	4.1 W/kg	8.2 W/kg	4.1 W/kg	10 W/kg	2 W/kg	0 W/kg
**Thermal**							
**classification**	**IV.**	**IV.**	**III.**	**I.**	**II.**	**I.**	**I.**

**IV.**  =  thermal; **III.**  =  slightly thermal; **II.**  =  most likely non-thermal; **I.**  =  non-thermal.

*) relating to the double-cone waveguide with only 1 of the 6 exposure places occupied.

**) Due to the varying results for different depths of the anaesthetical state only the range of the measured temperature elevation is given instead of mean and standard deviation.

***) determined from measured average initial brain temperature elevation via SAR  =  c (ΔT/Δt)|_t = 0_ with c = 3854 J/(kg K) the specific heat capacity of brain tissue.

### Plasma hormones

Hormonal mechanisms have been reported earlier to be in involved in learning and memory storage [28], [29]. Especially, hormones secreted from the HPA have been described to be highly implicated [30], [31], [32], [33]. In this context, appetitive and aversive learning have been shown to be influenced by the stress related hormones ACTH and CORT released after vegetative stimulation of the HPA [34]. Plasma CORT and ACTH elevations in association with stress are shown to provide major impact on LTP induction [18], [35], [36], [37], [38], [39], [40] and modification of LTD responses [41], [42], [43], [44], [45], [46] *in vivo* and *in vitro*.

To gain an impression whether changes in LTP and LTD after sham or EMF exposure are based exclusively on stress induced through the restraint conditions or whether a correlation to additive exposure induced effects exist, we analyzed basal plasma ACTH and CORT levels after finishing the individual experiments for each group. Levels in the HPA hormones of ED-only exposed animals and EMF exposed were compared to those of free running cage controls and to an additional group of handled animals.

### Plasma ACTH and CORT

**Figure 3:**
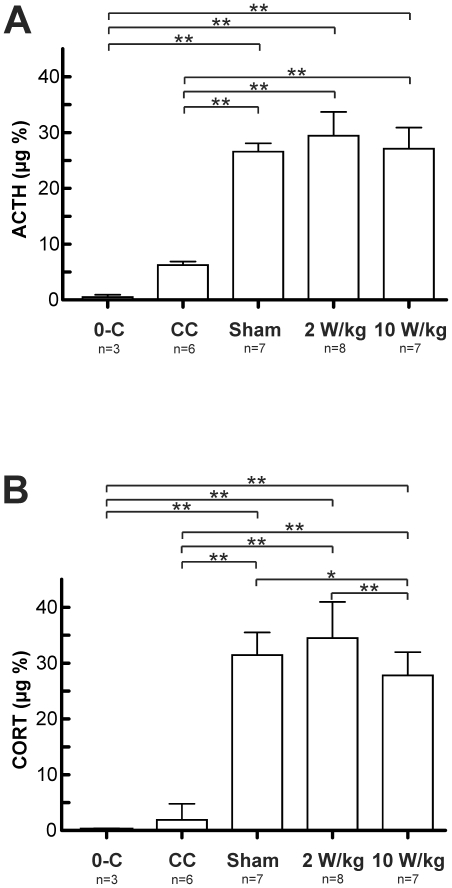
Measurements of the stress related plasma hormones. **3A–B** show the outcome of measurements of the stress related plasma hormones Adrenocorticotrophin (ACTH) and Corticosterone (CORT) in the differentially treated groups of animals. The blood was collected from the trunk after decapitation of the animals. Note, that a handled group (0-C) which was trained to visit the guillotine voluntarily after training was used for reference. **3A**, Holm-Sidak comparison of the ACTH levels revealed significantly increased values in cage controls (CC), the SAR 0 W/kg (Sham), 2 W/kg (2 W/kg) and 10 W/kg (10 W/kg) treated animals when compared with the 0-C group. Furthermore all exposure setup treated animals showed strongly increased ACTH levels when compared to the CC group, indicating a high degree of procedure related stress. Comparable findings were made in regarding the plasma CORT levels in **3B**. The result clearly underlines the handling and procedure related stress component.

The effect of ED-only and ED-EMF exposure on the plasma ACTH levels, as measured by radioimmunoassay is displayed in **Fig. 3A**. All animals following the 2 h lasting visit of the ED exhibited significantly increased basal ACTH (ANOVA: F_4, 27_ = 11.78; p<0.001) compared to cage- (CC) and handled controls (0-C) animals. Newman-Keuls post hoc analysis indicated that EMF exposed groups (2 W/kg and 10 W/kg) were significantly above the control groups (p<0.001). Statistical differences with correlation to the EMF strength were not observed among these groups (p_sham vs. 2 W/kg_ = 0.78; p_sham vs. 10 W/kg_ = 0.9; p_2 W/kg vs. 10 W/kg_ = 0.58).

Levels of CORT were seven to nine times higher in all ED-EMF exposed animals than those of cage- and handled control animals (**Fig. 3B**). The ANOVA of the data revealed a clear group effect (F_4, 27_ = 170.94; p<0.001). In this context, post-hoc Neuman-Keuls analysis indicated a generally significant (p<0.001) difference between the CORT levels of cage-control animals and those of the ED-EMF treated groups. Similar results were found for the comparison of the handled group to the latter ones (**Fig. 3B**). No differences were observed between both control groups (p = 0.42). Statistics of the EMF setup groups (sham, SAR 2 W/kg, SAR 10 W/kg) revealed significantly lowered CORT levels in the SAR 10 W/kg exposed group when compared to the SAR 2 W/kg (p<0.001) and the sham treated pool of animals (p<0.05).

These findings suggest that the animals are subject of a high acute stress impact during the visit of the ED in the restrained condition. The results are in accordance with observations described by Foy et al., 1987. The trend-like reduction in ACTH and CORT (**Fig. 3A and B**) in the presentiment free sacrificed handled group underline that handling and anaesthesia of the cage control group already initiates an acute stress reaction.

### Long term potentiation of fEPSP slopes

#### Group analyses

**Figure 4:**
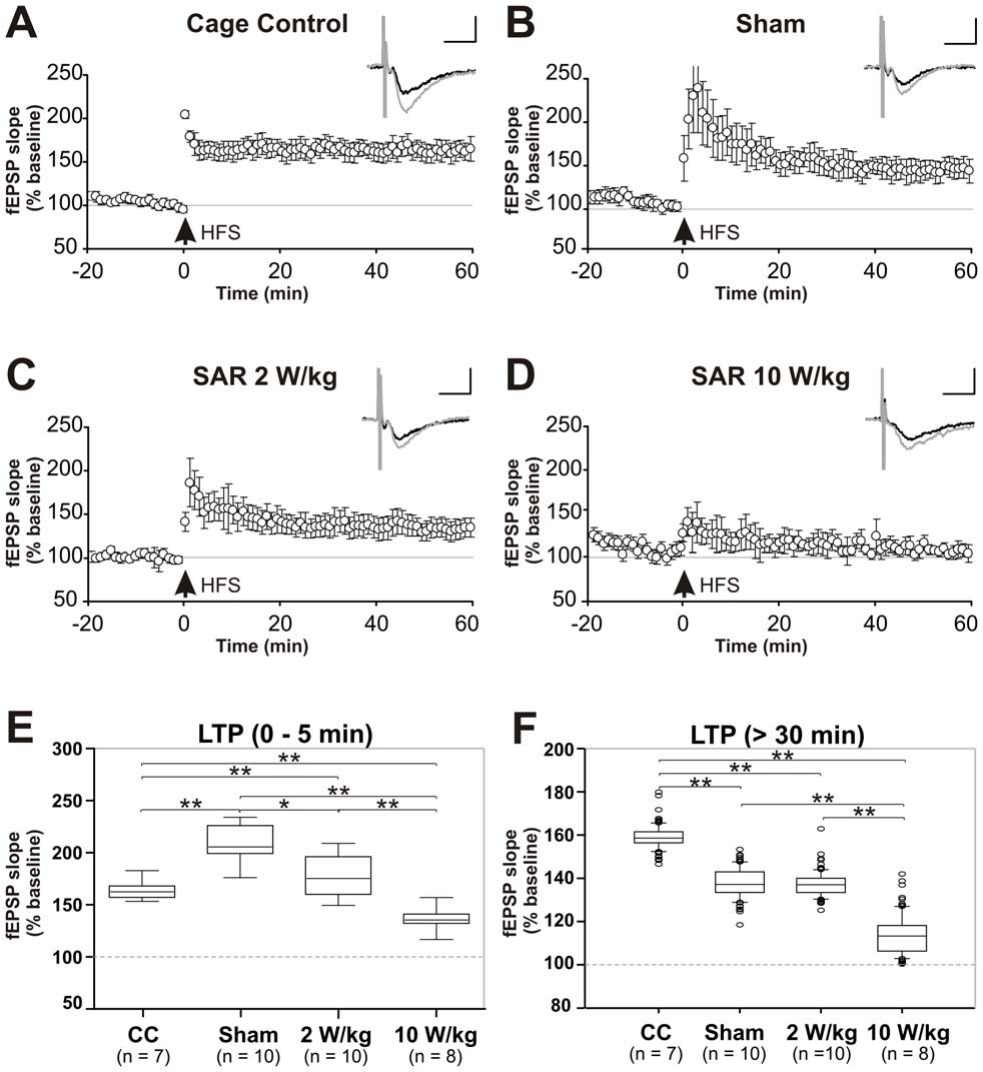
Induced CA1 long term potentiation (LTP) of field potential (fEPSP) slopes. **4A–F** LTP following high frequency stimulation (HFS) of the *Schaffer* collateral -commissural pathway of the Hippocampus in the differentially treated groups of Wistar rats. Reliable LTP of fEPSP slopes and amplitudes (as indicated in the figure insets) was achieved in 20–30 min after the theta burst and maintained for at least 40 min. The grey trace in the insets depicts the fEPSP 30 min post stimulation. In comparison to cage controls (**4A**) the Sham group (**4B**) as well as the SAR 2 W/kg exposed group (**4C**) were matter to pronounced early LTP during the first 20–30 min after HFS, followed by persistent LTP of low amplitude. Similar observations were made for the SAR 10 W/kg treated group (**4D**): HFS stimulation lead to a 30–40 min lasting phase of early LTP. In this case early LTP could not be shown to be converted into a subsequent phase of late LTP. Scale in insets: horizontal = 5 ms, vertical = 0.5 mV. In **4E**, fEPSP slopes between the four groups were compared during the first five minutes of early LTP induction. The process of early LTP is clearly reflected by the significant differences between the differentially treated groups. All exposure setup treated animals differed in their early LTP from the cage control group either in increased fEPSP slopes (Sham, 2 W/kg) or a lack in LTP induction (10 W/kg). Comparison of the late LTP phase >30 min post HFS in **4F** reveals significantly reduced fEPSP slopes in 10 W/kg exposed animals compared to all other groups.

A number of studies have shown that HFS *in vitro* and *in vivo* induce LTP maintaining for several hours [44], [47]. In the present study we recorded fEPSPs from the CA1 stratum radiatum of the hippocampus in response to stimulation of the *Schaffer* collateral-comissural pathway (**Fig. 1D**). After 20 min of stable baseline recording, LTP was induced by four sets of HFS at 100 Hz with 10 sec interset intervals. For data analysis of the fEPSP slopes (**Fig. 4** **)**, values derived from the last 10 min of baseline recording as well as from the period of 50–60 min of post HFS recording were used. In cage controls (**Fig. 4A**) we assessed robust LTP of extracellular field potentials. HFS succeeded in inducing reliable LTP of fEPSP slopes and amplitudes (as indicated in the figure insets) 20–30 min after the stimulation (n_CC_ = 7, 158.97±2.08% of baseline) and maintaining for at least 40 min. Compared to cage controls, the sham group (**Fig. 4B**) as well as the SAR 2 W/kg exposed group (**Fig. 4C**) were matter to pronounced early LTP during the first 20–30 min after HFS, followed by low amplitude late LTP (n_sham_ = 10, 137.87±2.16%; n_2 W/kg_ = 10, 137.26±1.82% of baseline). Different observations were made for the SAR 10 W/kg treated group (**Fig. 4D**): HFS stimulation also lead to a 30–40 min lasting phase of early LTP. But in this case early LTP could not be shown to be converted into a subsequent phase of late LTP (n = 8; 114.17±3.24% of baseline).

#### Intergroup comparison

The related boxplots in **Fig. 4E and F** of the slope values obtained from the recordings shown in **Fig. 4A–D** depict the differences between the inductive phase of the early LTP and the late LTP effects of the cage controls versus sham and EMF exposed animals. We used the first 5 min of the post HFS recording period for early LTP induction related data comparison (**Fig. 4E**). An ANOVA on the data in **Fig. 4E** revealed a main effect of groups (F_(3, 58)_  = 60,98; p<0.001). Holm-Sidak post-hoc comparison clearly indicates significantly increased fEPSP slopes for sham (p<0.001) and SAR 2 W/kg (p<0.001) exposed animal groups during the phase of LTP induction when compared to cage controls. fEPSP slopes during early early LTP induction in the 10 W/kg group were found to be generally reduced when compared to cage controls (p<0.001), sham (p<0.001) and SAR 2 W/kg (p<0.001) treated animals. Cage controls and sham exposed groups showed minor differences (p<0.04).

For evaluation of putative effects on persistent late LTP, the last 30 min of the complete post HFS recording period were used for data comparison in **Fig. 4F**. As indicated by the ANOVA analysis, there is a main effect on late LTP of the groups (F_3, 354_ = 615.12; p<0.001). Late LTP is impaired in all three EMF exposed groups in comparison to fEPSP slopes of cage controls (p_sham_ ≤0.001; p_2 W/kg_ ≤0.001; p_10 W/kg_ ≤0.001). Furthermore, 10 W/kg exposed animals were characterized by strongly reduced fEPSP amplitudes during persistent LTP when compared to sham exposed correlates (p_sham vs. 10 W/kg_ ≤0.001). It has to be noted that comparison of sham and 2 W/kg treated animals did not turn out to be significantly different (p_sham vs. 2 W/kg_ = 0.56). These data indicate that animals which have previously been exposed to ED-only for two hours are characterized by a general reduction in fEPSP slopes. SAR 10 W/kg treated animals, however, exhibit an additional significant affection of their HFS evoked LTP responses.

### Long term depression fEPSP slopes

#### Group analyses

**Figure 5:**
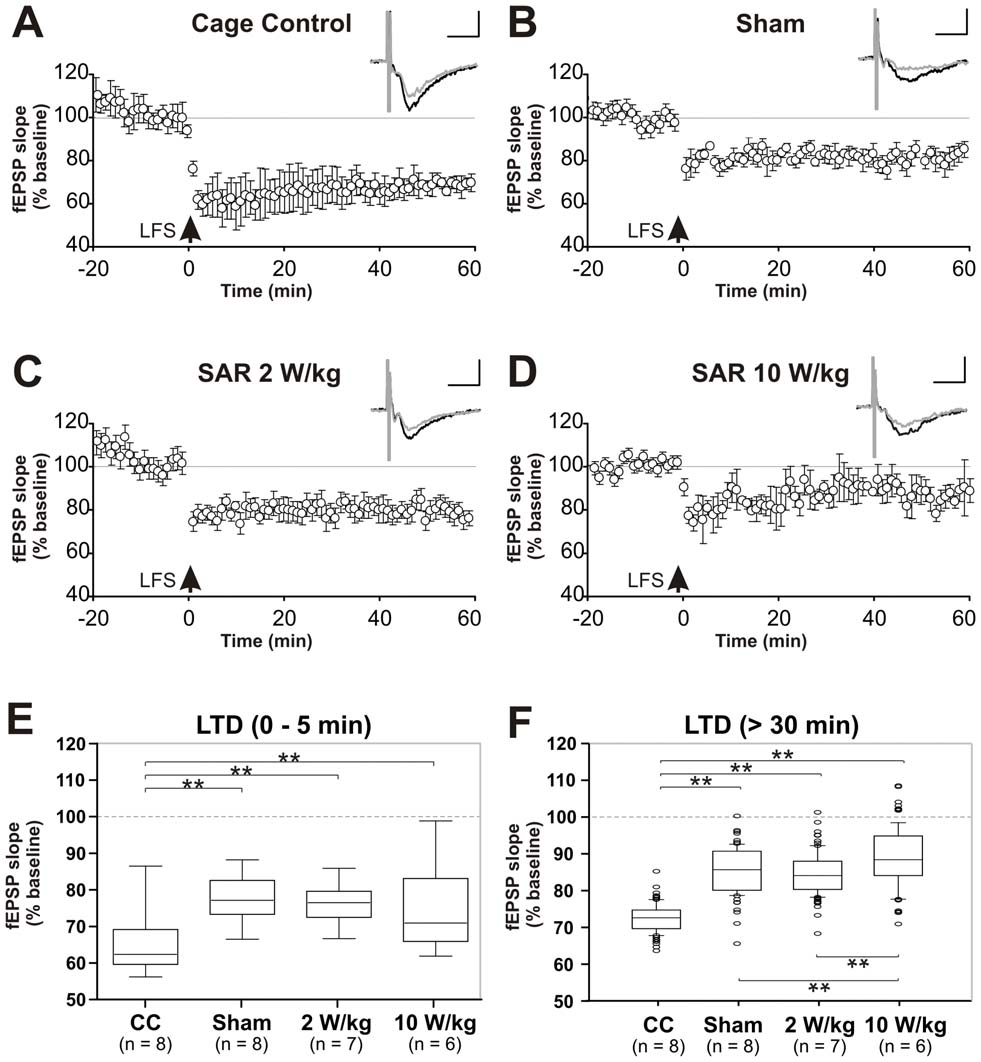
Induced CA1 long term depression (LTD) of field potential (fEPSP) slopes. **5A–F** LTD following low frequency stimulation (LFS) of the *Schaffer* collateral -commissural pathway of the Hippocampus in the differentially treated groups of Wistar rats. Sustained LTD of fEPSP slopes was induced in all four groups 20–30 min following LFS and at least maintained for 60 min. The grey trace in the insets depicts the fEPSP 30 min post stimulation. Compared to cage controls (**A**), Sham and EMF exposure treated animals (**B–D**) also showed LTD which was characterized by much lower changes in the LFS induced fEPSP responses. The SAR 10 W/kg treated group (**D**) was characterized by the lowest depression in fEPSP slopes 30 min post LFS. Scale in insets: horizontal = 5 ms, vertical = 0.5 mV. **5E** depicts the comparison of fEPSP slopes in the inductive phase of LTD during the first 5 min post LFS. As indicated by the figure insets in **5A–D**, all exposure setup treated animals were characterized by significantly flattened fEPSP slopes. This result became strengthened by the comparison of the fEPSP slopes 30 min after LTD induction in **5F**: All UMTS exposure setup treated animals exhibited a reduced LTD in which SAR 10 W/kg treated animals differed from the sham group by the weakest induced responses.

Next, we investigated the hippocampal LTD evoked in the recording by the stimulus electrode constellation as shown in **Fig. 1D**. Depression of extracellular field potentials was elicited in cage controls as well as sham and EMF treated groups after 20 min of stable baseline recording via low frequency stimulation (LFS) of the *Schaffer* collateral-comissural pathway. The stimulus protocol consisted of 900 pulses at 1 Hz. As illustrated in **Fig. 5A–D**, sustained LTD of fEPSP slopes were induced in all four groups 20–30 min following LFS, and maintained for at least 60 min. Compared to the exposure setup treated animals, cage controls exhibited the strongest reduction in fEPSP slopes (**Fig. 5A**) post HFS (n_CC_ = 8, 68.83±1.36% of baseline), maintaining for at least 60 min. Sham and EMF treated animals (**Fig. 5B–D**) also showed LTDs which were characterized by much lower changes in the LFS induced fEPSP responses than those observed in cage controls (n_sham_ = 8, 81.78±2.31%; n_2 W/kg_ = 7, 80.88±9.54% of baseline). As indicated in **Fig. 5D**, the SAR 10 W/kg treated group was characterized by the lowest depression in fEPSP slopes 30 min post LFS (n_10 W/kg_ = 6, 85.02±3.31% of baseline).

#### Intergroup comparison

The boxplots in **Fig. 5E and F** of the slope values obtained from the recordings depicted in **Fig. 5A–D** display the differences between the LTD effects in cage controls versus all ED exposed groups.

ANOVA analysis of the LTD inductive phases in **Fig. 5E** revealed a group effect (F_3, 46_ = 18.85, p<0.001). When compared to the cage control group, the Holm-Sidak method indicated a reduction in fEPSP slopes during LTP induction in all three EMF exposed groups, (p_sham_ <0.001; p_2 W/kg_ <0.001; p_10 W/kg_ <0.001). The latter groups did not differ among themselves.

A group effect on fEPSP slopes could also be detected via ANOVA during the 30–60 min period post LFS (F_3, 304_ = 82.26, p<0.001). Similar to the LTD induction, Holm-Sidak comparison clearly revealed significantly lower changes in fEPSP slopes for sham and EMF treated animals when compared to cage controls (p_sham_ <0.001; p_2 W/kg_ <0.001; p_10 W/kg_ <0.001). Although brain slices derived from sham and SAR 2 W/kg exposed animals showed a generally weakened LTD effect compared to the cage controls, both conditions did not differ in their slope response values (p_sham vs. 2 W/kg_ = 0.06). Similar to the effect already described for the induction of LTP, SAR 10 W/kg exposed animals responded in an essentially robust way and showed the lowest amount of LTD in comparison to the group of SAR 2 W/kg (p_2 W/kg vs. 10 W/kg_ <0.001) and sham exposed rats (p_sham vs._ _10 W/kg_ <0.001).

Thus the LTD data corroborate the observation made by the LTP experiments in the following ways: First, a 2 h stay in the ED system provides a counterproductive effect on hippocampal synaptic plasticity in the CA1 region which is also reflected by the post LFS fEPSP slope responses in the slices. Second, the SAR 10 W/kg exposed groups exhibit a significant effect on LTD generation which might be associated with the previous exposure of the animals to the EMF of the strongest absorption rate.

### Properties of basal synaptic transmission

**Figure 6:**
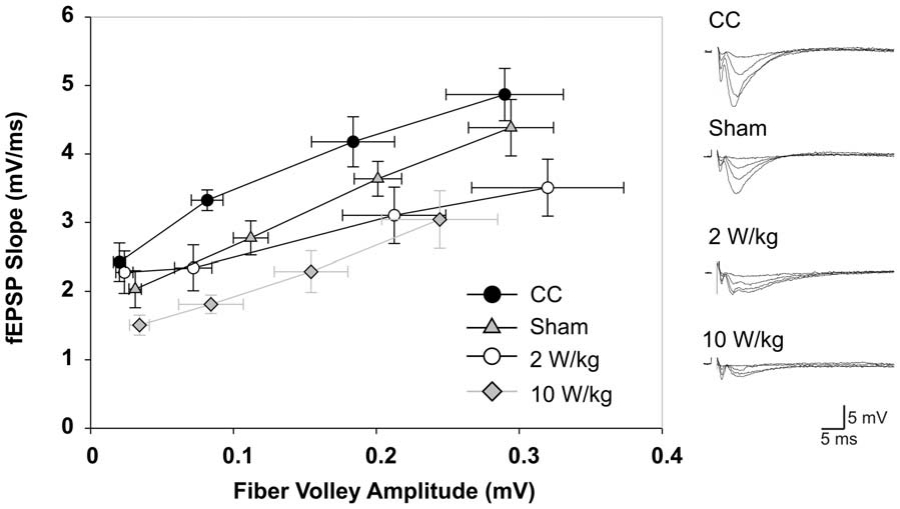
Comparison of the input/output function for basal synaptic transmission in slices. Brains were derived from cage controls and all three exposure setup treated groups. The size of presynaptic fibre volleys (input) was compared to the slopes of fEPSPs (output) measured from responses generated by different intensities (25, 50, 75, 100% of the maximum fEPSP amplitude) of presynaptic fibre stimulation. The 10 W/kg exposed group is characterized by a prominent left shift of their fibre volleys of the fEPSPs corresponding to 75 and 100% of the maximum fEPSP amplitude. Furthermore the group is characterized by general decrease in fEPSP slopes when compared to cage controls. These correlations underline the findings of the general negative influence of the exposure setup treatment on the animals' synaptic transmission properties which might be most probably due to exposure and restraint related stress.

To explore the putative effect of EMF exposure on basal synaptic function we compared the input/output function for basal synaptic transmission in slices from cage controls and the ED/ED-EMF groups. In these experiments the size of presynaptic fibre volleys (input) was compared to the slopes of fEPSPs (output) measured from responses generated by different intensities (25, 50, 75, 100% of the maximum fEPSP amplitude) of presynaptic fibre stimulation (**Fig. 6**). Slices of the 10 W/kg exposed group show a left shift of their fibre volleys of the fEPSPs corresponding to 75 and 100% of the maximum fEPSP amplitude, as can be depicted from **Figure 6**. Furthermore, this group is characterized by a general decrease in fEPSP slopes when compared to cage controls. These findings suggest an affection of the pre- and postsynaptic signalling properties in the SAR 10 W/kg exposed group. **Table 3** and **4** show the statistical differences after ANOVA and Holm-Sidak post-hoc analysis between all groups at the four stimulus intensities of the input/output correlations.

**Table 3:** Changes in fEPSP slopes.

Condition	25%	50%	75%	100%
CC vs. Sham	_(NS)_p = 0.16	_(NS)_p = 0.04	_(NS)_p = 0.04	_(NS)_p = 0.11
CC vs. 2 W/kg	_(NS)_p = 0.5	_(NS)_p = 0.02	_(NS)_p = 0.28	_(NS)_p = 0.43
CC vs. 10 W/kg	_(NS)_p = 0.07	** p = 0.007	** p = 0.007	* p≤0.05
Sham vs. 2 W/kg	_(NS)_p = 0.25	_(NS)_p = 0.13	_(NS)_p = 0.13	_(NS)_p = 0.14
Sham vs. 10 W/kg	** p = 0.003	** p≤0.001	** p≤0.001	** p≤0.001
2 W/kg vs. 10 W/kg	_(NS)_p = 0.04	** p = 0.002	** p = 0.002	** p = 0.004
ANOVA	F_3;44_ = 3.41;	F_3;44_ = 7.83;	F_0.35_ = 7.83;	F_3;44_ = 5.39;
	p = 0.025	p≤0.001	p≤0.001	p = 0.003

**Table 4:** Changes in presynaptic fiber volley amplitudes.

Condition	25%	50%	75%	100%
CC vs. Sham	_(NS)_p = 0.89	_(NS)_p = 0.83	_(NS)_p = 0.75	_(NS)_p = 0.79
CC vs. 2 W/kg	_(NS)_p = 0.21	_(NS)_p = 0.51	_(NS)_p = 0.02	_(NS)_p = 0.97
CC vs. 10 W/kg	_(NS)_p = 0.24	_(NS)_p = 0.05	** p = 0.009	** p = 0.005
Sham vs. 2 W/kg	_(NS)_p = 0.23	_(NS)_p = 0.32	_(NS)_p = 0.03	_(NS)_p = 0.8
Sham vs. 10 W/kg	_(NS)_p = 0.23	_(NS)_p = 0.04	** p≤0.001	** p≤0.001
2 W/kg vs. 10 W/kg	_(NS)_p = 0.22	** p = 0.009	** p≤0.001	** p≤0.001
ANOVA	F_3;56_ = 2.71;	F_3;56_ = 4.12;	F_3;56_ = 14.62;	F_3;56_ = 7.42;
	p = 0.053 (ns)	p = 0.01	p≤0.001	p≤0.001

ns  =  not significant.

It has to be noted that slices from the 10 W/kg treated group continuously differed from the cage controls and SAR 2 W/kg exposed animals by significantly decreased fEPSP slopes and presynaptic fibre volley amplitudes over a spectrum of three afferent stimulation intensities (50, 75, 100%). These data on basal synaptic properties suggest a link between restraint stress and glutamatergic synaptic transmission as observed in all other ED-only treated groups and underline an adverse effect for the SAR 10 W/kg group as additionally shown by the left shift of the curves in **Figure 6**. Such a shift has been described to be related to local activity dependent regulation of postsynaptic density proteins with impact on AMPA receptor recruitment and incorporation into excitatory synapses [48], [49], [50], [51], [52], [53], [54], [55], [56] and NMDA receptor [48], [56], [57], [58], [59], [60].

### Electromagnetic field effect or simply stress?

**Figure 7:**
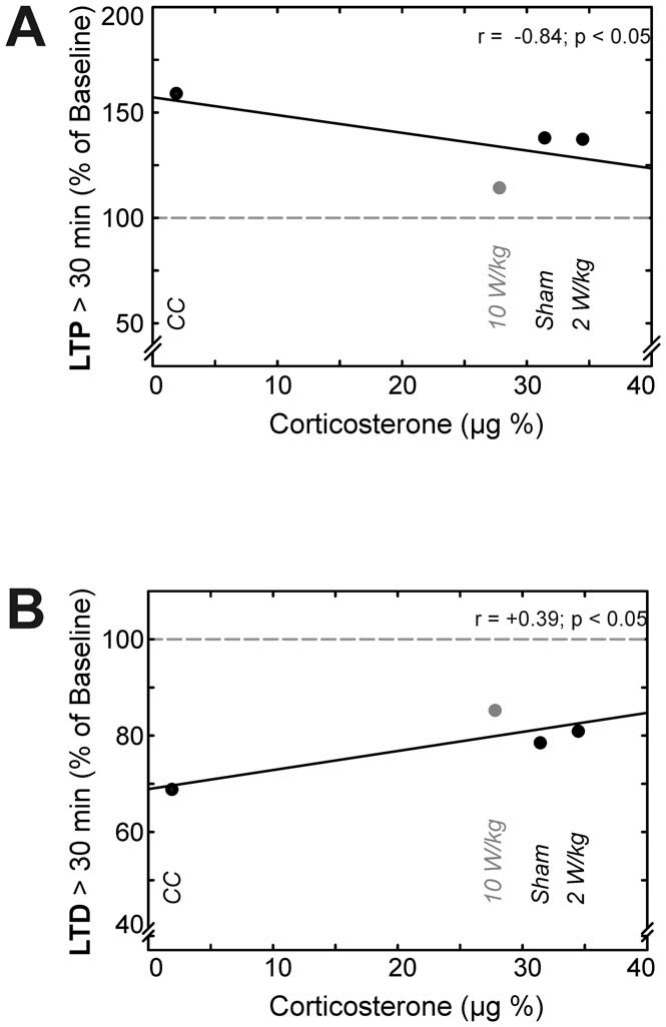
Correlation of stress hormone levels and synaptic plasticity. **7A**, levels in plasma corticosterone (CORT) were found to be negatively correlated with the outcome in late LTP of the four differentially treated groups (as indexed in the diagrams). CORT levels were positively correlated with persistent LTD of the groups in **7B**. Each data point combines the individual groups' mean CORT and fEPSP slope value for persistent late LTP/LTD.

To gain an idea about a possible coherence between HPA stress, EMF effect and synaptic plasticity in the hippocampal CA1 region, we calculated correlation coefficients between the persistent LTP/LTD (fEPSP slope as % of baseline) and levels in CORT in the differentially treated groups (**Fig. 7**). A negative correlation between plasma CORT and persistent LTP (r = −0.84; p<0.05) was obtained among the four groups (**Fig. 7A**). For persistent LTD (**Fig. 7B**), a positive correlation was found (r = +0.39; p<0.05). It has to be noted that the SAR 10 W/kg exposed group shows the most significant inhibition of synaptic plasticity in both correlations, although comprising the least CORT levels within the ED treated groups rendering an additive effect of the SAR 10 W/kg exposure to the restraint derived stress most likely.

## Discussion

This study reveals that restraint condition, as applied to sham and EMF exposure treated rats in a caged surrounding, elicits behavioural stress which is not only displayed by an increase of HPA hormones but also by changes in the electrically evoked synaptic responses of CA1 neurons after HFS or LFS of the *Schaffer* collateral commissural pathwa*y*.

Most importantly the sham and EMF exposure treated animals are characterized by impressive early LTP of their postsynaptic responses during the first 15 min post HFS. This finding closely correlates to recent observations of rapid and intense activation of endogenous plasticity in the hippocampus in the context of stressful experiences [35], [36], [37], [61]. Acute stress and subsequent upregulation in serum stress hormone levels is well described to profoundly alter certain forms of hippocampus dependent learning and memory [39], [40], [62], [63], [64]. Since Foy et al. (1987) described the influence of restraint stress on hippocampal neuronal plasticity, it is clear that the restraint conditions we applied in our experiments accounts for part of the neurophysiological changes obtained in our EMF setting. Although the animals stayed in the restraint condition within the ED for just 2 hours, it has been shown that acute inescapable stress can inhibit LTP in adult rats for up to 7 days [65], [66], [67]. For rat hippocampal slices Kohda et al. [68] showed that hippocampal learning and plasticity were still disrupted one week after a single prolonged stress.

These findings are in line with our observations that restrained sham and EMF treated rats exhibit smaller sized fEPSP responses with a decremental course after HFS compared to cage controls which were in general signed by non- decremental late LTP. The facilitated early LTP represents one of the key properties in fEPSP responses of sham and EMF exposed animals. Ryan et al. [69] evoked persistent stress in male rats by elevated platform exposure and were able to demonstrate a comparable inhibition of LTP subsequently and also 30 min post exposure.

In regard to EMF exposed animals, exposure to 2 W/kg elicited minor changes in fEPSP responses compared to the sham exposed group, however, SAR 10 W/kg completely inhibited generation of late LTP. Considering the equal baseline stress levels in all restrained groups, this finding clearly indicates that SAR 10 W/kg leads to an additive inhibition on hippocampal dependent plasticity. The mechanistic cues for this inhibition remain unclear.

All groups showed LTD responses of their fEPSP following 900 pulses of 1 Hz-stimulation of the *Schaffer* collateral- commissural fibre pathway. Similar to the outcomes of the LTP related experiments, ED and ED-EMF treated animals revealed lower LFS induced changes in their postsynaptic responses compared to the cage controls. Although there is an inhibition of late LTP in sham or EMF exposure treated animals, our experiments demonstrate that LTD is not facilitated in these groups.

We assume this effect to be closely related to the increase in basal ACTH and CORT. Although our post exposure CORT measurements revealed moderate serum concentrations of the stress related hormones, it has to be considered that levels of serum CORT reach their stress induced peak within the first 30 min of acute inescapable stress [43]. Therefore the peak of CORT concentration was already achieved at the beginning of the 2 hours lasting sham or EMF exposure period and the values obtained are already in the decaying phase.

Studies of Manahan-Vaughan and Braunewell [17] provided clear evidence that acute CORT elevations do not facilitate hippocampal CA1 LTD and suggest that a behavioural gating is needed for LTD. We assume the non-facilitation of the general LTD response of our sham or EMF exposure treated groups to be derived from the initial CORT elevation during the first 30 min of tube restraint within the ED. During all sham and EMF exposures animals were kept in a silent, equally illuminated and ventilated areal. This stimulus reduced condition did not provide the possibilities for novelty explorations as they are demonstrably needed to facilitate hippocampus-dependent LTP in a CORT level dependent manner [17]. Instead, behavioural gated facilitation of LTD is described as a multifactorial process which requires a concert of events like e.g. behavioural arousal, activation of the noradrenergic system [70], [71], [72] and activation of CORT receptors to modulate processes such as the modulation of beta-adrenoreceptors [73], to mention a few of them.

Whereas sham and SAR 2 W/kg exposed animals did not differ in their fEPSP responses after LFS, the SAR 10 W/kg treated group instead was characterized by the lowest LFS induced changes in the fEPSP response. This finding again underscores our interpretation of an adverse effect of the SAR 10 W/kg exposure to synaptic plasticity related phenomena.

The current descriptions of electromagnetic field effects on hippocampal neuronal plasticity in terms of LTP/LTD are sparse in the literature. However, there are reports that EMF effects might occur on cognitive functions and learning behaviour in human subjects or animals. Effects of endogenous and applied electric fields on the hippocampus and other structures are reviewed by Jeffreys [74].

In studies with human subjects, Preece and colleagues [75] used a copy of a commercial phone with an output of 1 W and showed that exposure to an analogue 915 MHz field for about 30 min lead to significant decreases in choice reaction time. No changes were observed with regard to simple reaction time, word, number, picture recall or spatial memory by use of GSM signals. Nevertheless, changes in choice reaction time were attributed to local tissue heating related increases in neuronal activity [76], [77].

Wang and Lai found, that exposure to 2.45 GHz fields for 1h also reduced spatial learning in behavioural studies in the rat [78]. A potential physiological mechanism to explain these effects was reported by [79]. Here, the authors describe that direct exposure of acutely dissected hippocampal brain slices to non-thermal 700 MHz wave radiofrequency fields can modulate the excitability of the hippocampal tissue. As a possible explanation of the observed effects of modulated pyramidal cell activity, the authors discuss the induction of weak electric fields within the tissue, in turn influencing local neuronal excitability.

An ultimate explanation of the underlying cellular mechanism remains still enigmatic. A recent report by Anastassiou et al. [80] points into a direction which should be further perused. The authors describe neuronally induced extracellular field fluctuations responsible for ephaptically (non-synaptic) mediated changes in the somatic membrane potentials of neurons. Despite their small size, these fields were able to strongly entrain action potentials. They conclude that such endogenous brain activity can causally affect neural functioning through electric field effects. It thus awaits to be further explored whether externally applied electromagnetic fields might interfere with this internal local field potentials and to which end neuronal activities might be affected.

### General implications

The results of our sham and EMF exposed animals are clearly influenced by an obvious stress effect, which is in line with the current literature. Nevertheless, our study reveals evidence that: **a**) Full head sham or SAR 2 W/kg exposure of adult male Wistar rats can be regarded as harmless in terms of hippocampus-dependent neuronal plasticity. **b**) Full head exposure of the rats to SAR 10 W/kg leads to significant non-thermal affection of hippocampus dependent LTP and LTD. Since Varro et al. (2009) also showed a decrease in basic synaptic activity after EMF exposure of hippocampal and cortical slices *ex vivo* (250–500 µT, 50 Hz), it has to be assumed that the SAR 10 W/kg effect is reproducible under isolated slice conditions.

Nevertheless, higher-powered UMTS exposure is correlated with significant shifts in synaptic long term plasticity in rats. These effects appear in addition to the procedure related stress and can be correlated with the UMTS exposure procedure. The results provide the new finding that long term activity-dependent synaptic plasticity is a putative target for the effect of electromagnetic fields of extraordinary high energy absorption rates in male adult Wistar rats.
